# Cross-Shore and Depth Zonations in Bacterial Diversity Are Linked to Age and Source of Dissolved Organic Matter across the Intertidal Area of a Sandy Beach

**DOI:** 10.3390/microorganisms9081720

**Published:** 2021-08-12

**Authors:** Julius Degenhardt, Julian Merder, Benedikt Heyerhoff, Heike Simon, Bert Engelen, Hannelore Waska

**Affiliations:** 1 Institute for Chemistry and Biology of the Marine Environment, University of Oldenburg, 26111 Oldenburg, Germany; julius.degenhardt@uni-oldenburg.de (J.D.); benedikt.heyerhoff@uni-oldenburg.de (B.H.); heike.simon@uni-oldenburg.de (H.S.); hannelore.waska@uni-oldenburg.de (H.W.); 2 Department of Global Ecology, Carnegie Institution for Science, Stanford, CA 94305, USA; jmerder@carnegiescience.edu

**Keywords:** microbial diversity, dissolved organic matter, DOM, functional diversity, sandy beach, intertidal area, North Sea, SGD

## Abstract

Microbial communities and dissolved organic matter (DOM) are intrinsically linked within the global carbon cycle. Demonstrating this link on a molecular level is hampered by the complexity of both counterparts. We have now investigated this connection within intertidal beach sediments, characterized by a runnel-ridge system and subterranean groundwater discharge. Using datasets generated by Fourier-transform ion cyclotron resonance mass spectrometry (FT-ICR-MS) and Ilumina-sequencing of 16S rRNA genes, we predicted metabolic functions and determined links between bacterial communities and DOM composition. Four bacterial clusters were defined, reflecting differences within the community compositions. Those were attributed to distinct areas, depths, or metabolic niches. Cluster I was found throughout all surface sediments, probably involved in algal-polymer degradation. In ridge and low water line samples, cluster III became prominent. Associated porewaters indicated an influence of terrestrial DOM and the release of aromatic compounds from reactive iron oxides. Cluster IV showed the highest seasonality and was associated with species previously reported from a subsurface bloom. Interestingly, Cluster II harbored several members of the candidate phyla radiation (CPR) and was related to highly degraded DOM. This may be one of the first geochemical proofs for the role of candidate phyla in the degradation of highly refractory DOM.

## 1. Introduction

Dissolved organic matter (DOM) is one of the major carbon sources within the oceans and an active compartment of the global carbon cycle [1]. In the water column, it is produced and released by phytoplankton [2], making it one of the main nutrient sources for heterotrophic bacteria in marine environments [3]. Another large source of chemically distinct DOM is terrestrial runoff through rivers and groundwater into the coastal ocean [4]. The release of terrestrial and marine DOM from rivers and coastal sediments, together with inorganic nutrients like dissolved iron and ammonium, has been shown to fuel phytoplankton blooms, thus transforming terrestrial inorganic and organic matter into marine algal-produced DOM [5,6]. Labile, low molecular weight compounds like phytoplankton-derived organic matter (OM) are degraded preferentially. In comparison, high molecular weight plant polymers, e.g., cellulose or lignin, accumulate as specialized enzymes are necessary for their degradation [7]. It is estimated that 90% of the oceans’ carbon pool is refractory dissolved organic carbon [8]. The most stable compounds within that pool form the so-called island of stability with a turnover time of geological timescales [9]. Despite the methodological challenges, the composition and fate of DOM has been investigated in many aquatic environments like oceans, rivers, and estuaries [10,11,12,13] as well as the porewaters of terrestrial and marine environments [14,15,16]. The fate of DOM in subterranean estuaries (STEs) and porewaters of intertidal areas is of special interest, since here terrestrial and marine components are mixed, substantially altered, and potentially released again into the coastal ocean [17]. These processes take place along the flow paths of STEs and the so-called intertidal seawater recirculation cell [6,18]. At sandy and high energy beaches, the remineralization is additionally enhanced due to elevated recharge of oxic seawater and organic matter by strong physical forcing. In combination with advective porewater flow, both lead to enhanced rates of an initial aerobic degradation of DOM [18,19]. In previous studies on porewaters of North Sea tidal flat margins [20] or a sandy beach aquifer [21], it was found that those sediments are also sources of recalcitrant and dissolved back carbon (DBC) for the coastal ocean. Based on the composition of inorganic constituents, this discharge was linked to anoxic processes, yet overlooking the microorganisms driving those processes.

Heterotrophic bacteria in marine sediments not only depend on the import of fresh DOM from the water column [22], but out of necessity, many are also capable of degrading older and more recalcitrant DOM [23,24]. Their potential to degrade OM depends on environmental factors like electron acceptor availability [25], quantity and quality of OM [26], and a wide variety of other factors such as temperature-driven kinetics and porewater residence time [27]. Furthermore, their physiological capability of OM degradation is determined by functional traits like (exo)enzymes necessary to break down larger polymers [28,29] and the pathways to degrade a certain substrate spectrum. Phyla like the Flavobacteria and Planctomycetes can usually degrade a wide range of polymers [30], while groups like Gammaproteobacteria and Deltraproteobacteria are known for their capability of fermentation or using small volatile fatty acids, respectively [31,32,33]. A group of organisms probably involved in the degradation of highly recalcitrant DOM is the “candidate phyla radiation group” (CPR group). They are found in a wide range of habitats from freshwater [34] and marine environments [35,36], but also the terrestrial realm [37] and aquifers [38,39]. They are supposed to be obligate symbionts or syntrophs due to their lack of essential biosynthetic genes [38,40]. Nevertheless, metagenomic evidence suggests that they are capable of degrading complex DOM like cellulose, starch, and lipopolysaccharides [41]. By supplying degradation products like acetate to the surrounding microbial community, they might have a “priming” effect on carbon cycling within their respective environment [41,42].

Despite the obvious link between microorganisms and DOM cycling, only a few studies have investigated the mutual influence of DOM and bacterial diversity so far. For instance, correlations between labile DOM and the active part of bacterial communities were detected within the coastal North Sea [43]. Those correlations could be subtle due to steady state substrate conditions within the water column. However, heterotrophic bacteria likely play a major role in the production of recalcitrant DOM [44]. In another study by Oni et al. [45], algal polymer-associated microbial communities were detected in muddy surface sediments of Helgoland (Germany), while deeper sediments were dominated by microbes known for degrading more recalcitrant DOM. They could also observe a correlated shift in DOM composition from oxygen rich aromatics with a low hydrogen-carbon ratio and highly unsaturated molecules to compounds containing less oxygen while having a higher hydrogen to carbon ratio. Based on those findings, we aimed to expand this knowledge by investigating DOM cycling in sediment porewaters and connecting it with bacterial community structures and predicted metabolic functions.

As a model study site, we chose a high-energy beach in the German North Sea. Previous studies found indications of rapid and efficient organic matter degradation, resulting in the accumulation of inorganic nutrients and recalcitrant DOM along the advective porewater flow paths [19,46,47]. Studies on microbial diversity revealed a large core community as well as cross-shore gradients across the intertidal area [19,48,49]. However, diversity data only allow speculations about functional diversity, making it difficult to establish links between microbial metabolism and molecular traits in DOM. By additionally using the bioinformatic tool Tax4Fun2, it is possible to infer functions from 16S rRNA gene amplicon data based on metagenomic reference databases [50]. Hypothetical functional profiles are partly comparable to metagenome sequencing and allow for the generation of a nuanced artificial dataset to characterize functional properties of the microbial communities. In this study, we applied this novel data processing tool, and furthermore used a conditional inference tree to determine the factors responsible for the observed community compositions.

The main questions we wanted to answer were: (i) Are there patterns in DOM composition and functional community profile across the intertidal area? If so, (ii) can we detect relationships between bacterial diversity, their metabolic potential, and the DOM composition, gaining insight into the processes dominating DOM remineralization within the beach sediments? To do so, we used 16S rRNA gene amplicon data, predicted functions, and linked them to a DOM dataset obtained by FT-ICR-MS. The analysis spans the upper meter of sediment, sampled along two transects across the intertidal area at three different seasons.

## 2. Materials and Methods

### 2.1. Site Description

Spiekeroog Island is part of the East Frisian Barrier Island chain located in the southern North Sea, with the German Wadden Sea in its back barrier region. The island harbors a subsurface freshwater lens, which is separated from sea water by a density gradient and which partially flows northward to form a subterranean estuary (STE) facing the open North Sea. The intertidal area of this STE is characterized as a mesotidal, sandy, high energy beach with a runnel-ridge system. This system is frequently relocated within the intertidal area due to tides, waves, and storm surges. At elevations above the mean sea-level, seawater infiltrates into the sediments, while areas below are net submarine groundwater discharge (SGD) areas of the STE [51]. Due to the strong beach morphodynamics, in- and exfiltration areas are not static. Thus, the discharge is patchier than observed for other STEs. Detailed descriptions of hydrogeochemistry, geochemistry and microbial diversity can be found in previous studies investigating the Spiekeroog STE [19,52,53].

### 2.2. Sediment Sampling and Porewater

Samples were collected in October 2016, March 2017 and August 2017 at the seaward beach of Spiekeroog Island. Sediment cores were recovered with aluminum core liners along two transects between the high (HWL) and low water line (LWL). Four cores were taken along each transect at specific topographical features: HWL, runnel, ridge, and LWL. During the first sampling campaign in October 2016, an additional sediment core was retrieved at the LWL (LWLb). Due to the tidal range, this site was not accessible during the following sampling campaigns. All cores were subsampled at 0–2, 10–12, 30–32, 50–52, and 100–102 cm depth using sterile cut-off syringes. Samples were stored frozen at −20 °C until further processing as previously described in Degenhardt et al. [48].

Porewater samples were obtained by using stainless steel push-point samplers and pre-rinsed polyethylene syringes and were processed according to Ahrens et al. [46] for O_2_, NO_3_^−^, NO_2_^−^, NH_4_^+^, Fe, and Si. PO_4_^³−^ was determined photometrically, following the methods of Itaya and Ui [54] for samples with concentrations below 2.1 µM and Laskov et al. [55] for samples exceeding 2.1 µM PO_4_^³−^. Porewater samples used for DOC analysis were treated and processed according to Waska et al. [53], and DOM samples were extracted and analyzed with ultra-high resolution Fourier-transform ion cyclotron resonance mass spectrometry (FT-ICR-MS) according to Waska et al. [47]. In addition to the chemical characteristics (e.g., elemental ratios and compound classes) and molecular indices (e.g., Ideg and ITerr), which were calculated in the same way as in Waska et al. [47], we included here the homologous series network number, which is described in Merder et al. [56] as part of the ICBM-OCEAN data processing output. DNA extraction was performed with Phenol chloroform after a modified protocol of Lueders et al. [57] and Gabor et al. [58]. PCR as well as sequencing settings can be found in Degenhardt et al. [48].

### 2.3. Bioinformatics and Statistical Analysis of Sequence Data

Raw sequence data were processed as described in Degenhardt et al. [48]. In short, dissimilarities between samples were calculated using the Bray-Curtis dissimilarity applied on Hellinger transformed bacterial read counts [59]. Hierarchical clustering using the complete linkage algorithm was performed on the resulting dissimilarity matrix to cluster samples based on similar community composition [59,60]. The optimal number of clusters was five based on the silhouette coefficient, which quantifies the quality of clustering achieved. The silhouette coefficient is based on the arithmetic mean of silhouette values, which are calculated for each sample and represent how well a sample fits into its attributed cluster compared to the next neighboring cluster. Thus, with this analysis, not only the optimal cluster number can be identified but also the strength of each sample to belong to its cluster. As cluster V represents only one sample, it was excluded for the following interpretation, and the subsequent analysis was based on the four remaining clusters. For each of the clusters, bacterial species that are representative for their respective cluster were identified based on the indicator species value (IndVal) [61]. This value is calculated for each species-cluster combination and is defined as the product of the fidelity of a species (average relative abundance of species_j_ that are in a cluster_k_ divided by the sum of average relative abundances of species_j_ in all other clusters) and specificity of a species (proportion of samples in the cluster_k_ that contain the species_j_). Statistical significance of the IndVal for a species cluster combination is based on permutation tests. The *p*-values were corrected for multiple testing based on the Benjamini-Hochberg method [62]. Only significant (*p_adj_* < 0.05) zOTUs were considered in the follow-up analysis and interpretation [62]. To link the DOM composition to the observed bacterial clusters, we also repeated the IndVal analysis on Hellinger transformed data of intensities of molecular formulae identified in DOM.

We fitted a Conditional Inference Tree to explain the observed clustering by chemical and metabolic covariates [63]. Those covariates included chemical parameters such as O_2_, NO_3_^−^, NO_2_^−^, NH_4_^+^, Fe, Si, PO_4_^³−^, and DOC, as well as the predicted presence of genes indicative for, e.g., nitrification, denitrification, sulfate reduction, or polymer degrading enzymes. A Conditional Inference Tree is a class of decision tree that applies binary splits at thresholds of explanatory variables to lead to an optimal separation between clusters. The threshold and explanatory variable that leads to the lowest *p*-value based on a permutation test statistic is selected at each split. If no further splitting increases the separation into clusters significantly (here *p* ≥ 0.001), splitting stops. Results are visualized as a binary tree. Because we know how well a sample fits into its respective cluster based on the silhouette value calculation, we used this value as additional weighting in the Conditional Interference Tree analysis.

### 2.4. Functional Prediction Using Tax4Fun2

Functional prediction based on the 16S rRNA genes was carried out using Tax4Fun2 [50] and R (version 4.0.3) available online: http://www.R-project.org (accessed on 10 October 2020). Tax4Fun2 relies on the SILVA [64] and KEGG [65] databases and associated 16S rRNA genes with specific KEGG orthologues and pathways. Tax4Fun2 was able to use on average 4.998% (Cluster I), 4.305% (Cluster II), 4.957% (Cluster III), and 39.912% (Cluster IV) of all sequences to a functional profile representing 3.463%, 3.147%, 4.957%, and 12.702% of all zOTUS respectively. The low prediction efficiencies are probably related to the usage of all zOTUs also uncultivated or unknown, considerably lowering the prediction efficiency. The output was plotted in heat maps using the R packages “ggplot2” [66] and “gridExtra” [67]. Hereby, we focused on key genes, which are frequently used to detect metabolic pathways within environmental samples, since they encode for enzymes catalyzing a specific reaction by microbial cells. The entire list of genes that were screened can be found in the heat maps within the Supplementary Material Figures S2–S8.

## 3. Results

### 3.1. Clusters in Community Composition

**Figure 1:**
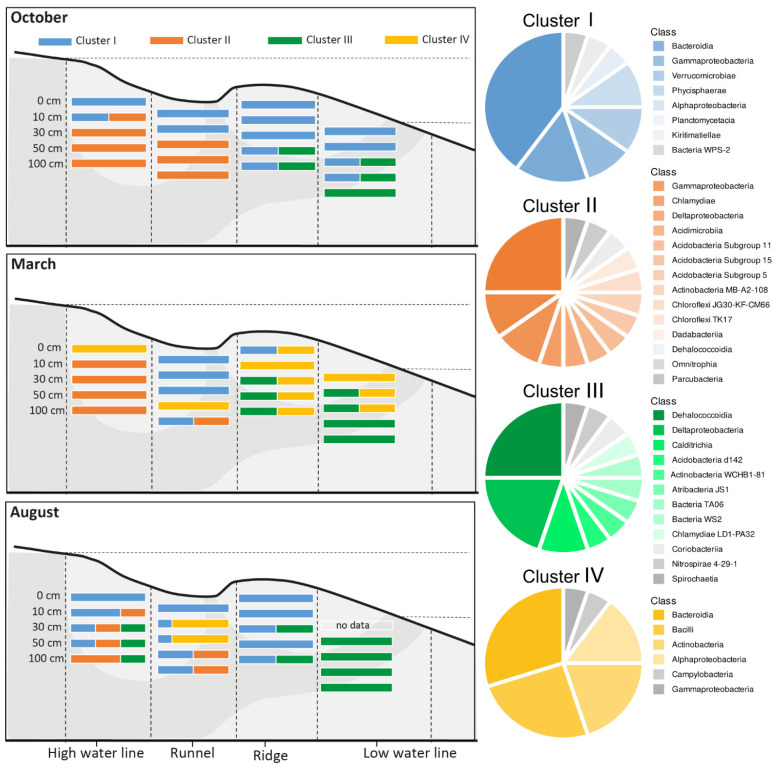
The spatial and temporal distribution of the four clusters based on their relative abundances within the Illumina dataset. The pie charts depict the 20 most representative classes of each cluster based on the respective IndVals. A NMDS visualizing the taxonomic relationship between the cluster can be found in the Supplementary Figure S1.

The community composition of the investigated beach has previously been described as very homogeneous. We achieved a more detailed insight by using a statistic approach to find the optimal number of clusters within the bacterial communities that describes its composition. Four clusters with an individual composition and distribution across the intertidal zone of Spiekeroog beach were identified (Figure 1). Based on Indicator species values (IndVal), cluster I was composed of 68 zOTUs (IndVal 0.73–0.32), cluster II of 153 zOTUs (IndVal 0.95–0.17), cluster III again of 68 zOTUs (IndVal 0.92–0.14), and cluster IV of 36 zOTUs (IndVal 0.92–0.17). We only considered IndVals that are significantly high after *p*-value adjustment. Focusing on the 20 “best” hits based on their IndVal, cluster I was dominated by Flavobacteria (*n* = 9), the other groups present being Planctomycetes (*n* = 3), Alphaproteobacteria (*n* = 2), Gammaproteobacteria (*n* = 3), Verrucomicrobia (*n* = 2), and Kirimatellaeota (*n* = 1). Cluster II was not only the largest but also most heterogeneous group with Acidobacteria (*n* = 3), Chloroflexi (*n* = 1), Actinobacteria (*n* = 2), Gammaproteobacteria (*n* = 4), Deltaproteobacteria (*n* = 1) Chlamydiae (*n* = 3), Myxococcales (*n* = 1), the candidate phyla Candidatus Yanofskybacteria, Candidatus Omnitrophus, and Candidatus Protochlamydia. Cultivated closest relatives of the latter are known as endosymbionts of amoeba [68].

Even though the used primers did not specifically target Archaea, reads of Nitrosopumilus zOTUs were associated with cluster II. This is plausible since reads of Nitrosococcus zOTUs also fell into cluster II, while being not among the top 20. Cluster III also contained three different archaeal zOTUs. However, all archaeal zOTUs will be neglected in the following analysis, since the primers used were not designed to target Archaea and their detection was due to a miss-priming during sequencing. The dominant bacterial groups were Chloroflexi (*n* = 6), and also present were Deltaproteobacteria (*n* = 3), Sphingomonadales (*n* = 1), Chlamydia (*n* = 1), Actinobacteria (*n* = 2), Acidobactereia, Spirotriches, and Nitrospirae with one zOTU each. It is worth noting that all Chloroflexi detected belong to the Dehalococcoides. Cluster IV was the least diverse with Flavobacteria (*n* = 4), Actinobacteria (*n* = 6), Alphaproteobacteria (*n* = 4), and Firmicutes (*n* = 6). All firmicute related zOTUs were members of the Planococcaceae, and all zOTUs related to Alphaproteobacteria were members of the Roseobacter group. Despite some overlapping taxa, the four clusters were taxonomically different, as can be seen in NMDS plot Figure S1 within the supplements.

zOTUs of cluster I were present at every site in October. This was also the case during August, except for the LWL samples. In March, they were present only at the ridge and runnel. Overall, this cluster was strongly represented in samples from 0 cm and 10 cm but could be detected at a deeper depth as well, depending on site and season. Cluster II increased in relative abundance at deeper depths of the HWL and runnel sites and was especially pronounced at the HWL but was not detected at the ridge and LWL. Instead, cluster III showed elevated relative abundances from 30 cm downwards at the ridge and particularly the LWL site. Its members increased in relative abundances in March and especially August, where the LWL was completely characterized by cluster III, and it could even be detected at the HWL from 30 cm downwards. Cluster IV was not representative for any October sample but was very dominant in March, especially at the ridge and LWL, but also at the surface of the HWL. In August, this cluster was only found in larger relative abundances at 10 cm and 30 cm at the runnel site. Similar to cluster II, it also showed no distinct depth related pattern.

### 3.2. DOM Components Related to Clusters within the Bacterial Community

**Figure 2:**
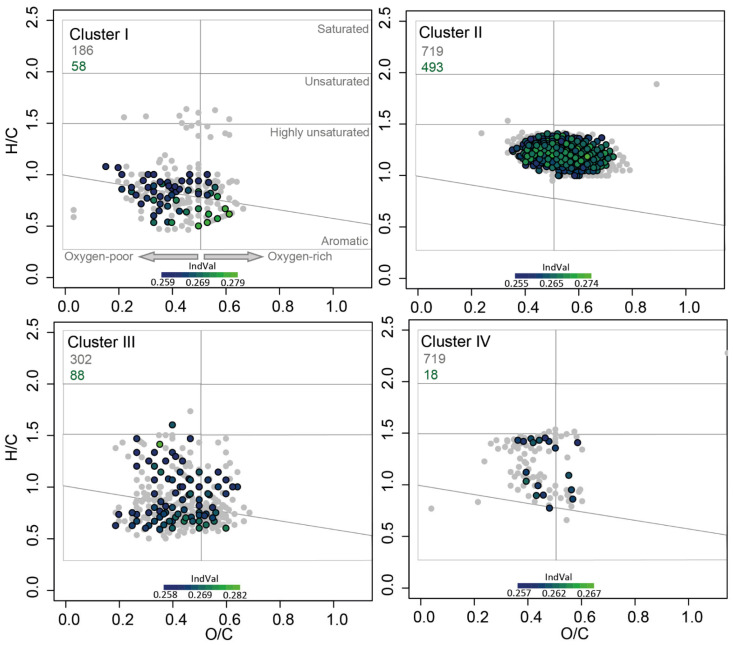
Van Krevelen plots of all DOM compounds linked to one of the four bacterial clusters. Grey dots indicate masses correlated, but not significant after *p-adjustment*. Colored dots are also correlated significantly after *p*-adjustment. Grey numbers give the amount of related masses, colored numbers the proportion that is significant after *p*-adjustment. The color scale represents IndVal, with higher IndVals indicating stronger correlations.

In a next step, we identified masses within the porewater DOM pool that were significantly related to each of the four clusters derived from bacterial beta-diversity (Figure 2). DOM clusters corresponding to those diversity clusters were characterized by very similar H/C (mean 1.15 to 1.17) and O/C ratios (mean 0.47). In the van Krevelen space, cluster II related compounds were located in the so called “island of stability” and comprised of masses that are very resistant to degradation. It also showed almost no scatter. Cluster I and III had a similar amount of significantly correlated masses with cluster III related molecules showing a scatter towards higher H/C ratios. Cluster IV had the lowest number of significantly related masses. Some of those clustered at the boundary between unsaturated and highly unsaturated oxygen poor compounds and showed a scatter towards a higher degradation state.

Porewater and DOM constituent characteristics related to the four clusters showed strong heterogeneity in some cases, since they included seasonal and spatial variations. The comparison of the highest and lowest mean values nevertheless revealed some interesting trends. Cluster I related masses had the highest mean concentrations of porewater DOC and Mn together with the highest average nitrogen heteroatoms (N), aromaticity index (AI.mod), double bond equivalents (DBE), and aromatic compounds (Table 1). Furthermore, the related DOM compounds had the lowest average H/C and O/C ratios, molecular mass (Da), homologous series, and highly unsaturated compounds. Geochemical porewater conditions related to cluster II displayed the highest salinity, oxygen, and nitrate concentrations, as well as the lowest ammonium, silicate, iron, manganese, DOC, and FDOM concentrations. DOM compounds related to this cluster had the highest amount of highly unsaturated formulae, mean O/C ratio, homologous series members, and the lowest AI.mod. In contrast to this, the highest average ammonium, silicate, iron, and FDOM concentrations and the lowest salinity, oxygen, and nitrate concentrations were associated with cluster III. The related DOM compounds were characterized by the lowest mean O/C ratios (shared with cluster I), unsaturated compounds, DBE, and nitrogen heteroatoms. Cluster IV was characterized chemically by the highest average H/C ratio, molecular mass (Da), and amount of sulfur heteroatoms.

Using the whole DOM pool of sites that classed in each cluster, we calculated molecular indices indicative of degradation state (Ideg and MLB_l, [69,70]) and terrestrial influence (ITerr, [71]). Cluster I associated sampling sites showed the highest average molecular lability index (MLB_1) in their DOM geometabolome that represents the entirety of DOM produced by biotic and abiotic processes. In contrast, cluster II-associated sites displayed the lowest MLB_l, the highest mean degradation index (Ideg), and the lowest terrestrial index (ITerr). For cluster III, the highest ITerr was found, while Ideg was among the lowest. No significant trends were apparent for molecular indices calculated from cluster IV-associated sites.

### 3.3. Predicted Functional Diversity and Its Influence on Cluster Formation

**Figure 3:**
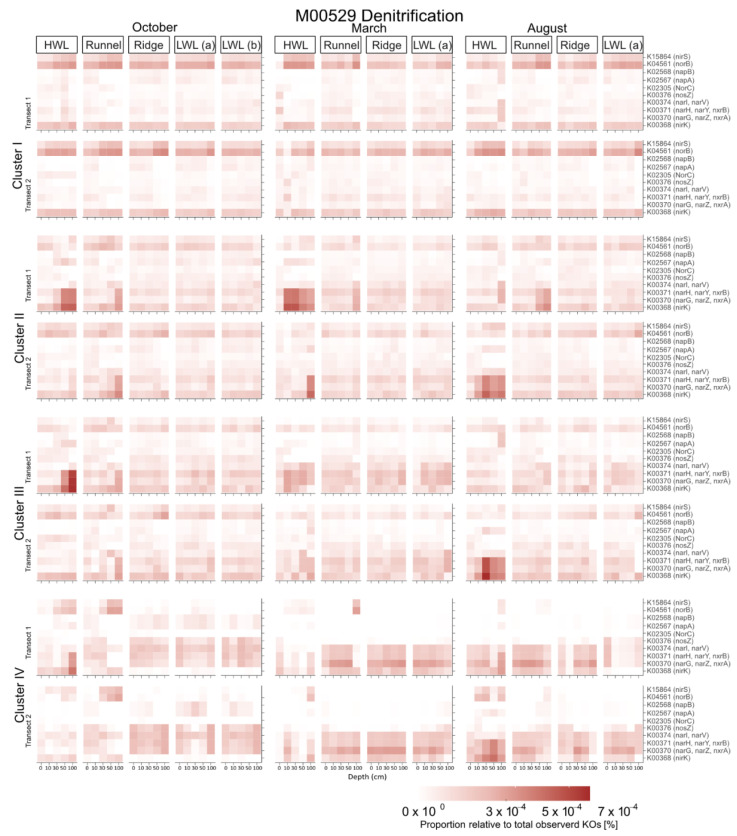
Predicted expression of genes involved in denitrification in relation to all KEGG orthologs (KOs) observed. Gene expression levels are presented individually for each of the four clusters at every station, depth, and month across the two transects sampled.

For an insight beyond the community compositional level, we created artificial metagenomes by predicting functions based on 16S rRNA gene data and corresponding KEGG orthologs. We specifically screened for the presence of key genes for catabolic processes, such as nitrate and nitrite reduction (Figure 3), ammonia oxidation, nitrogen fixation, sulfate reduction, and sulfur oxidation. Furthermore, we looked for genes related to polysaccharide degradation with a special focus on algal polymers (alginate, laminarin, fucoidan, carrageenan) but also polymers of terrestrial origin like starch, cellulose, tannin, and lignin. All genes that were predicted in notable amounts were used within a decision tree. For a complete overview of predicted genes screened, see Supplementary Materials Figures S2–S8.

**Figure 4:**
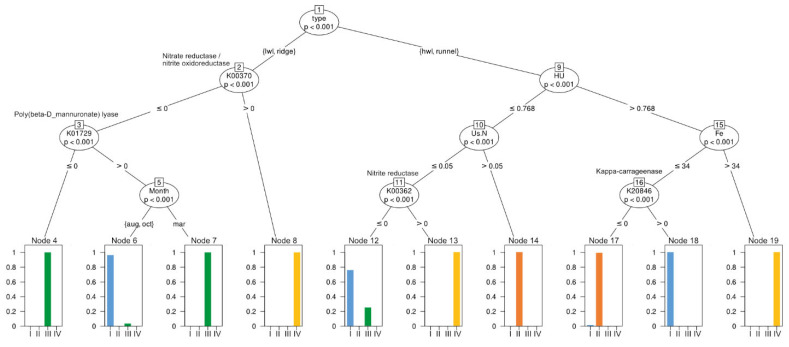
Conditional inference tree. Porewater nutrient data, DOM data and indices, and key genes of catabolic processes were used for a prediction of which environmental factors favor the occurrence of the bacterial clusters detected. Cluster I = blue, Cluster II = orange, Cluster III = green, Cluster IV = yellow.

In order to assign potential ecological roles to the detected clusters, we combined the predicted functional genes together with DOM indicator species as well as environmental and nutrient data into a decision tree (Figure 4). The presence of cluster I was positively related to the predicted presence of polymannuronate lyase at the LWL and ridge during August and October, or the presence of K-carrageenase at the HWL and runnel, when porewater DOM was already more degraded (highly unsaturated (HU) > 0.768) and dissolved iron concentrations were comparably low (≤34 µM). While cluster I was linked to splits caused by the predicted absence of nitrite and nitrate reductase, cluster IV was always related to the presence of both enzymes. Cluster IV was also linked to a higher HU index and higher dissolved iron concentrations (>34 µM), which might indicate an additional ecological role unrelated to denitrification. As previously described, cluster II was exclusively found at the upper beach sites, HWL and runnel. The only tested parameters with a relation to cluster II were unsaturated nitrogen-rich DOM compounds and a HU index < 0.768. If this index was higher, lower dissolved iron concentration and the absence of K-carrageenase were predicted to favor the presence of cluster II. The occurrence of cluster III was also unrelated to the parameters tested, such as the presence of nitrate and nitrite reductase or polymannuronate lyase. The predicted presence of the latter was the only link to cluster III, but exclusively within samples taken in March, which might be indicative for seasonal changes in algal polymer supply.

## 4. Discussion

In this study, we used next generation sequencing data as well as FT-ICR-MS data to investigate relations between microbial and chemical diversity. We were able to detect a significant relation between 30–500 DOM compounds with each of the clusters identified within beta-diversity. Those clusters do not represent the most abundant zOTUs, but are significantly linked to distinct zones within the intertidal area. For a deeper insight, we applied advanced bioinformatic approaches and additionally predicted functional diversity.

### 4.1. Depth and Cross-Shore Distribution of Bacterial Clusters Is Governed by DOM Age and Source

The four clusters detected within the bacterial community composition showed distinct horizontal and vertical distributions across the intertidal area. According to our predicted functional data and previous studies, aerobic organic matter degradation of algal polymers by cluster I bacteria, and nitrate reduction especially by cluster IV, seem to dominate shallower sediments and seasonal dynamics across the intertidal area. The latter is in accordance to findings by Ahrens et al. [46]. In contrast, cluster II and III were never representative for the surface samples (0 cm) and often increased in relative abundance, or even dominated deeper depth. Notably, cluster II was exclusively found at the HWL and runnel sites, while cluster III was almost exclusively found at the ridge and LWL. Furthermore, cluster II and III showed no link to any of the investigated functional genes but were linked to the degradation state of DOM.

This distribution is presumably related to observable changes within geochemical conditions along the flow path of the intertidal recirculation cell, most likely caused by different sources and residence times of porewater [19,51,52,53]. The degradation state and composition of DOM which influence the presence of cluster II and III at Spiekeroog beach are in turn influenced by the mixing of the terrestrial and marine endmembers. The first is characterized by containing old and partially plant-derived constituents [21,53] while the latter is characterized by less degraded, algae-derived nitrogen-rich DOM [4]. Both are mixed within the seawater recirculation cell and degraded along the flow path of the STE. Thus, our data support previous findings [7,27,72] that microbial and molecular diversity are linked by mutually influencing each other.

### 4.2. Candidate Phyla and the Island of Stability

Cluster II was linked to the largest amount of DOM compounds, which were also forming the so-called “island of stability” within the van Krevelen space (Figure 2). The island of stability was first observed as an accumulation of recalcitrant open-ocean DOM, which may resist microbial degradation up to millennial timescales [73]. However, similar trends in DOM fractionation were reported from incubation experiments with river and lake water [74,75]. It appears that during degradation, molecular formulae in DOM progress towards the island of stability. However, the large amount of related masses is probably also caused by the characteristics of cluster II, which is the largest and phylogenetically most heterogeneous of all four clusters. The porewater data that are related to cluster II were characterized by the highest average salinity, oxygen, and nitrate concentrations, which is indicative of seawater infiltration and recirculation areas. The DOM within those samples was on average composed of the largest compounds with the highest O/C ratio and degradation index. The latter fits to the presence of this cluster in deeper depths.

Interestingly, we detected eleven so-far uncultured members of the CPR-group (three among the 20 best fitting zOTUs) and the island of stability to be associated to cluster II. The CPR-group is almost exclusively known from metagenomic studies and is hypothesized to be involved in the degradation of highly recalcitrant OM and partially in nitrate reduction [40,41,76,77]. By doing so, they might provide easier degradable DOM to the surrounding community, possibly enhancing microbial carbon turnover rates. The Gammaproteobacteria associated to cluster II are likely some of these community members benefitting from such DOM upcycling [31]. These hypotheses from studies by Danczak et al. [77] and Vigneron et al. [41] are now backed up by our environmental and community data for the first time.

Another adaptation to recalcitrant DOM is the presence of many zOTUs related to chemolithoautotrophic bacteria such as Sulfuristfustis or Candidatus Omnitrophus [78,79]. Those organisms are suspected or known to produce H_2_, which would additionally supply electron donors to those bacteria already benefitting from the potential priming effect of CPR-group members. Another conspicuity within the composition of cluster II is the presence of Chlamydiae. Many of those are intracellular parasites of Acantamobae, including Candidatus Protochlamydiae [68]. Nevertheless, a recent study by Dharamshi et al. [80] found a wide variety of non-host associated Chlamydiae in deep-sea sediments. Therefore, chlamydial zOTUs within cluster II might also be non-host associated but free living within the sediment matrix.

### 4.3. SGD Impacted Sediments Are Characterized by a Typical Deep Subsurface Community

Porewater samples that are linked to cluster III were characterized by the highest ammonium, silicate, dissolved iron, and FDOM concentrations, which is in line with their link to the lower beach (ridge, LWL). This can especially be explained by the LWL being an exfiltration area of the subterranean estuary fed by recirculating seawater and fresh groundwater from the island’s freshwater lens [51,53]. On average, the DOM compounds associated with cluster III had the lowest degradation index and DBE but highest ITerr. The high ITerr as well as the relatively low salinity revealed an enhanced influence of terrestrial, vascular plant-derived DOM, connected to cluster III. This is in accordance with findings by Seidel et al. [21] and Waska et al. [53] who described an export of terrestrial compounds and DBC along the STE into the coastal North Sea. Furthermore, vascular plant-derived DOM strongly interacts with reactive iron oxides, resulting in adsorption under oxic, and release under reducing conditions [73,81,82]. Significant positive correlations of humic-like FDOM concentrations with those of dissolved iron have been reported as an indication that a substantial fraction of FDOM interacts with iron cycling [47,82]. Such a geochemical link would explain the co-occurrence of high FDOM and iron concentrations related to cluster III.

The relatively high aromaticity of DOM is reflected in the bacterial community composition, since cluster III was dominated by members of Dehalococcoida and Deltaproteobacteria. The latter include the typical sulfate-reducing bacteria of anoxic marine sediments, which preferentially make use of small carbon compounds. Even though cluster III contained several Deltaproteobacteria, sulfate reduction does only play a minor role at the depths investigated in our study. Nevertheless, cluster III related molecular formulae had elevated relative sulfur contributions and the highest number of S-containing formulae among all clusters (Table 1). Analogous to intertidal flats in the Wadden Sea, sulfurization of DOM is likely to take place at or below the low water line, albeit to a much lesser extent [20]. Dehalococcoida, in turn, are also known for the anaerobic degradation of halogenated and aromatic compounds [83,84,85,86,87]. Furthermore, cultivated strains are known to require hydrogen or formate as an electron donor and acetate in order to produce biomass [88]. Apart from that, Dehalococcoides have been detected in deep sediments by many previous investigations [24,85,86,89]. A study on mudflat sediments close to Helogoland by Oni et al. [45] also detected related sequences within the deep parts of their sediment cores (3–5 m). Furthermore, the authors found a connection between their occurrence and older, more recalcitrant DOM, which is in accordance with our findings. Since those sediments are much deeper and not known to be impacted by SGD, the dominance of Chloroflexi in our samples is probably more related to the anoxic subsurface conditions than to the freshwater influence.

### 4.4. Algal Polymer Degraders and the Lack of Algal Polymers

Cluster I was detected at every site and distributed across all depths, yet showed a strong seasonal variation. Nevertheless, especially in October and August, it dominated shallower samples (0–10 cm). Since cluster I contained a large amount of flavobacterial and planctomycete zOTUs, we identified it as “the surface cluster”. The community is mostly thriving under oxic conditions and probably involved in the degradation of algal-derived DOM [90,91]. The presence of cluster I at deeper depths of the ridge and LWL in October or at the HWL, runnel, and ridge in August could be attributed to sediment burial by physical reworking. Additionally, the presence of facultative anaerobes such as Chloroflexi, of the genus Phycisphaera within cluster I, supports its connection to algal biomass, since cultivated representatives of that genus have been detected on algal tissue [92]. This is backed up by porewater data related to cluster I, which had on average the highest values for DOC concentrations, as well as MLB_l, and the amount of N-containing molecular formulae. The high abundance of nitrogen heteroatoms fits to algal derived biomass, which has been described to be nitrogen-rich [4]. The connection between the presence of cluster I to the predicted presence of alginate lyase and K-carrageenase additionally suggests a role in degrading algal polysaccharide polymers under mostly oxic conditions. The high DBE could therefore be caused by the degradation of alginate into guluronic acid and mannuronate. The increase in double bonds is even used in quantification of alginate degradation [93]. Note that we could not detect molecular formulae of any of the oligomers, i.e., the building blocks of alginate or k-carrageenan in our DOM pool. Although the BOND ELUT PPL cartridges were developed for the isolation of compounds with a wide polarity range, the SPE method still has lower recoveries for highly polar compounds compared to those with a more hydrophobic character [94]. Moreover, due to the fast turnover of mono- and oligomeric sugars and amino acids, they are already scarce in natural aquatic systems. Easily degradable labile DOM is rapidly taken up and degraded under oxic conditions, which may lead to an underrepresentation of those compounds within the dataset, in addition to the semi-selectivity of the analytical approach. Contradictory to our assumption of cluster I to be connected to the degradation of algal polymers, cluster I related DOM was also characterized by containing on average the smallest compounds and the highest amount of aromatic compounds. This and the comparably high Ideg of this surface-associated cluster might be explained by the few DOM data from a 0 cm depth due to porewater scarcity.

### 4.5. Predicted Functional Data Support the Hypothesis of a Subsurface Bloom of Denitrifiers

Cluster IV showed no spatial pattern but a temporal pattern by appearing in March in high relative abundances and still being visible at the runnel in August. This observation has been described previously in a preceding publication by Degenhardt et al. [48]. The clear connection of cluster IV to the predicted presence of nitrate reductase at the LWL and ridge within the decision tree analysis supports the previous findings where the subsurface bloom was related to enhanced concentrations and penetration depths of nitrate during that month. The additional connection to higher HU and dissolved iron concentration allows a speculation concerning an involvement of some Cluster IV members in iron cycling when nitrate and labile OM are not available. While in the preceding study, the subsurface bloom was attributed to members of the Planococcaceae as well as Gillisia, we also found a large amount of zOTUs related to Actinobacteria and Alphaproteobacteria within that cluster. Since Alphaproteobacteria of the roseobacter group are also known to be opportunistic regarding their substrate spectrum, their contribution to cluster IV fits with previous findings by Buchan et al. [90]. Most zOTUs related to the Actinobacteria were members of the genus Nocardioides, which includes many cultivated representatives involved in denitrification. Thus, their presence is also in line with the hypothesis of a subsurface bloom of denitrifiers [48].

## 5. Conclusions

The synergistic analysis of bacterial and DOM diversity in combination with predictions of metabolic functions as well as applying advanced statistical methods revealed previously overlooked relations of both counterparts. We could detect a relation between four bacterial clusters and associated porewater DOM compounds which showed distinct cross-shore and depth zonations. Within deep upper beach sediments, members of the candidate phyla radiation, ultra-small Gammaproteobacteria, and several autotrophic bacteria are linked to DOM compounds of the island of stability. This finding supports a previously published metagenomic hypothesis about the involvement of candidate phyla in the degradation of very recalcitrant DOM which might then be utilized by other bacteria. SGD-impacted deep sediments of the lower beach showed a correlation of small, partially terrestrial-derived DOM compounds and Dehalococcoides, which are typically found in deep sediments and are also suspected to be involved in the degradation of recalcitrant DOM. The relation between nitrogen-rich DOM, Flavobacteria, and the predicted presence of algal polymer degrading enzymes indicates an involvement of the surface community in alginate and carrageen degradation across the intertidal area. Lastly, we could confirm our previous hypothesis about a subsurface bloom of denitrifiers with the help of predicted functional data. Furthermore, the findings are a promising starting point for future metagenomic and transcriptomic studies.

## Figures and Tables

**Table 1:** Average (± standard deviation) concentrations of environmental parameters and relative abundances of DOM molecular characteristics for the four clusters. DOM molecular data were derived from intensity weighted means of molecular formulae with a *p*_adj_ < 0.05 (depicted in van Krevelen plot). The highest value of each parameter amongst the four clusters is in bold and the lowest in italic. Wilcoxson pairwise comparisons between the clusters were performed for each category; the results are included in Supplementary Table S1.

	Cluster I	Cluster II	Cluster III	Cluster IV
Salinity	29.86 ± 3.59	**30.21 ± 2.83**	*27.43 ± 6.65*	29.18 ± 2.49
O_2_ (µM)	20.63 ± 40.73	**101.32 ± 122.48**	*13.11 ± 26.96*	46.01 ± 53.33
NH_4_ (µM)	16.81 ± 15.46	*3.17 ± 3.85*	**24.73 ± 22.25**	11.03 ± 11.97
NO_3_ (µM)	6.77 ± 13.61	**13.71 ± 15.8**	*6.41 ± 17.54*	9.99 ± 16.66
Si (µM)	49.81 ± 27.35	*34.05 ± 16.01*	**92.38 ± 57.31**	49.1 ± 18.65
Fe (µM)	29.59 ± 40.18	*0.16 ± 0.19*	**36.96 ± 35.19**	18.28 ± 30.73
Mn (µM)	**10.69 ± 15.54**	*0.74 ± 1.82*	5.59 ± 6.06	3.91 ± 3.89
DOC (µM)	**136.08 ± 29.95**	*105.49 ± 20.66*	128.5 ± 19.02	131.25 ± 21.08
FDOM (ppb QSE)	41.47 ± 10.65	*29.65 ± 10.71*	**50.51 ± 10.26**	38.37 ± 10.99
Homologous series	*8326 ± 251*	**13039 ± 81**	12925 ± 55	11835 ± 98
H/C ratio	*0.82 ± 0.01*	1.21 ± 0.003	0.99 ± 0.01	**1.24 ± 0.02**
O/C ratio	*0.41 ± 0.01*	**0.53 ± 0.003**	*0.41 ± 0.003*	0.46 ± 0.003
N	**1.23 ± 0.06**	0.21 ± 0.02	*0.02 ± 0.002*	0.17 ± 0.01
S	*0*	0.005 ± 0.0004	0.13 ± 0.01	**0.19 ± 0.01**
CHO	10 (17)	**314 (68)**	59 (67)	*9 (50)*
CHON	*48 (83)*	**141 (30)**	*4 (5)*	*4 (22)*
CHOS	*0 (0)*	9 (2)	**25 (28)**	5 (28)
Mass (Da)	*294.25 ± 1.85*	464.37 ± 1.88	315.01 ± 2.01	**488.55 ± 1.25**
AI.mod	**0.58 ± 0.01**	*0.23 ± 0.002*	0.45 ± 0.01	0.24 ± 0.01
DBE	**10.01 ± 0.06**	9.48 ± 0.04	*9.18 ± 0.16*	9.92 ± 0.2
Aromatic	**72.45 ± 1.71**	*0*	37.34 ± 2.63	4.11 ± 0.76
Highly Unsaturated	*27.55 ± 1.71*	**100 ± 0**	62.08 ± 2.60	95.89 ± 0.76
Unsaturated	0	0	**0.58 ± 0.09**	0
Unsaturated with N	0	0	0	0
Saturated	0	0	0	0
* Ideg	*0.70 ± 0.07*	**0.79 ± 0.09**	*0.70 ± 0.05*	0.72 ± 0.07
* MLB_l	**9.22 ± 1.66**	*8.63 ± 2.28*	8.73 ± 1.47	9.18 ± 1.63
* ITerr	0.21 ± 0.02	*0.17 ± 0.02*	**0.22 ± 0.02**	0.20 ± 0.02

Numbers of respective molecular formulae in each cluster with a *p*_adj_ < 0.05, with percent of total in brackets. * The molecular indices Ideg, MLB_L, and ITerr were calculated using the whole DOM datasets from the sites which were associated with each of the clusters (all seasons combined). Information on compound classes can be found in the text as well as in Waska et al. [47].

## Data Availability

16S sequencing data have been deposited to be publicly accessible at the European Nucleotide Archive (ENA) under the project accession number PRJEB39926. Geochemical data used for this study are available at PANGAEA under https://doi.org/10.1594/PANGAEA.905928.

## Supplementary Materials

The following are available online at https://www.mdpi.com/article/10.3390/microorganisms9081720/s1, Figure S1: NMDS of all Hellinger standardized zOTUs of the respective clusters. Colors indicate cluster affiliation, distance between datapoints indicates taxonomic dissimilarity. Figure S2: Predicted expression of genes involved in assimilatory nitrate reduction in relation to all KEGG orthologs (Kos) observed. Gene expression levels are presented individually for each of the four clusters at every station, depth, and month across the two transects sampled, Figure S3: Predicted expression of genes involved in dissimilatory nitrate reduction to ammonium in relation to all KEGG orthologs (Kos) observed. Gene expression levels are presented individually for each of the four clusters at every station, depth, and month across the two transects sampled, Figure S4: Predicted expression of genes involved in assimilatory sulfate reduction in relation to all KEGG orthologs (Kos) observed. Gene expression levels are presented individually for each of the four clusters at every station, depth, and month across the two transects sampled, Figure S5: Predicted expression of genes involved in sulfur oxidation in relation to all KEGG orthologs (Kos) observed. Gene expression levels are presented individually for each of the four clusters at every station, depth, and month and month across the two transects sampled, Figure S6: Predicted expression of genes involved the degradation of algal-derived polymers in relation to all KEGG orthologs (Kos) observed. Gene expression levels are presented individually for each of the four clusters at every station, depth, and month across the two transects sampled, Figure S7: Predicted expression of genes involved in dissimilatory sulfate reduction in relation to all KEGG orthologs (Kos) observed. Gene expression levels are presented individually for each of the four clusters at every station, depth, and month across the two transects sampled, Figure S8: Predicted expression of genes involved in nitrification in relation to all KEGG orthologs (Kos) observed. Gene expression levels are presented individually for each of the four clusters at every station, depth, and month across the two transects sampled. Table S1: Wilcoxson pairwise comparisons between environmental factors and DOM indices of each cluster. *p*-values are given for each significant difference.

## References

[1] Ridgwell A., Arndt S. (2015). Why Dissolved Organics Matter. Biogeochemistry of Marine Dissolved Organic Matter.

[2] Thornton D.C.O. (2014). Dissolved organic matter (DOM) release by phytoplankton in the contemporary and future ocean. Eur. J. Phycol..

[3] Azam F., Fenchel T., Field J.G., Gray J.S., Meyer-Reil L.A., Thingstad F. (1983). The Ecological Role of Water-Column Microbes in the Sea. Mar. Ecol. Prog. Ser..

[4] De Leeuw J.W., Largeau C. (1993). A Review of Macromolecular Organic Compounds That Comprise Living Organisms and Their Role in Kerogen, Coal, and Petroleum Formation. Organic Geochemistry. Topics in Geobiology.

[5] Avery G.B., Willey J.D., Kieber R.J., Shank G.C., Whitehead R.F. (2003). Flux and bioavailability of Cape Fear River and rainwater dissolved organic carbon to Long Bay, southeastern United States. Glob. Biogeochem. Cycles.

[6] Avery G.B., Kieber R.J., Taylor K.J., Dixon J.L. (2012). Dissolved organic carbon release from surface sand of a high energy beach along the Southeastern Coast of North Carolina, USA. Mar. Chem..

[7] Mahmoudi N., Beaupre S.R., Steen A.D., Pearson A. (2017). Sequential bioavailability of sedimentary organic matter to heterotrophic bacteria. Environ. Microbiol..

[8] Hansell D.A., Carlson C.A., Schlitzer R. (2012). Net removal of major marine dissolved organic carbon fractions in the subsurface ocean. Glob. Biogeochem. Cycles.

[9] Lechtenfeld O.J., Kattner G., Flerus R., McCallister S.L., Schmitt-Kopplin P., Koch B.P. (2014). Molecular transformation and degradation of refractory dissolved organic matter in the Atlantic and Southern Ocean. Geochim. Cosmochim. Acta.

[10] Kim S., Simpson A.J., Kujawinski E.B., Freitas M.A., Hatcher P.G. (2003). High resolution electrospray ionization mass spectrometry and 2D solution NMR for the analysis of DOM extracted by C18 solid phase disk. Org. Geochem..

[11] Koch B.P., Witt M., Engbrodt R., Dittmar T., Kattner G. (2005). Molecular formulae of marine and terrigenous dissolved organic matter detected by electrospray ionization Fourier transform ion cyclotron resonance mass spectrometry. Geochim. Cosmochim. Acta.

[12] Tremblay L.B., Dittmar T., Marshall A.G., Cooper W.J., Cooper W.T. (2007). Molecular characterization of dissolved organic matter in a North Brazilian mangrove porewater and mangrove-fringed estuaries by ultrahigh resolution Fourier Transform-Ion Cyclotron Resonance mass spectrometry and excitation/emission spectroscopy. Mar. Chem..

[13] Sleighter R.L., Hatcher P.G. (2008). Molecular characterization of dissolved organic matter (DOM) along a river to ocean transect of the lower Chesapeake Bay by ultrahigh resolution electrospray ionization Fourier transform ion cyclotron resonance mass spectrometry. Mar. Chem..

[14] Einsiedl F., Hertkorn N., Wolf M., Frommberger M., Schmitt-Kopplin P., Koch B.P. (2007). Rapid biotic molecular transformation of fulvic acids in a karst aquifer. Geochim. Cosmochim. Acta.

[15] Schmidt F., Elvert M., Koch B.P., Witt M., Hinrichs K.-U. (2009). Molecular characterization of dissolved organic matter in pore water of continental shelf sediments. Geochim. Cosmochim. Acta.

[16] Longnecker K., Kujawinski E.B. (2011). Composition of dissolved organic matter in groundwater. Geochim. Cosmochim. Acta.

[17] Santos I.R., Burnett W.C., Dittmar T., Suryaputra I.G.N.A., Chanton J. (2009). Tidal pumping drives nutrient and dissolved organic matter dynamics in a Gulf of Mexico subterranean estuary. Geochim. Cosmochim. Acta.

[18] Reckhardt A., Beck M., Seidel M., Riedel T., Wehrmann A., Bartholomä A., Schnetger B., Dittmar T., Brumsack H.-J. (2015). Carbon, nutrient and trace metal cycling in sandy sediments: A comparison of high-energy beaches and backbarrier tidal flats. Estuar. Coast. Shelf Sci..

[19] Beck M., Reckhardt A., Amelsberg J., Bartholomä A., Brumsack H.-J., Cypionka H., Dittmar T., Engelen B., Greskowiak J., Hillebrand H. (2017). The drivers of biogeochemistry in beach ecosystems: A cross-shore transect from the dunes to the low-water line. Mar. Chem..

[20] Seidel M., Beck M., Riedel T., Waska H., Suryaputra I.G.N.A., Schnetger B., Niggemann J., Simon M., Dittmar T. (2014). Biogeochemistry of dissolved organic matter in an anoxic intertidal creek bank. Geochim. Cosmochim. Acta.

[21] Seidel M., Beck M., Greskowiak J., Riedel T., Waska H., Suryaputra I.G.N.A., Schnetger B., Niggemann J., Simon M., Dittmar T. (2015). Benthic-pelagic coupling of nutrients and dissolved organic matter composition in an intertidal sandy beach. Mar. Chem..

[22] Jorgensen B.B., Boetius A. (2007). Feast and famine--microbial life in the deep-sea bed. Nat. Rev. Microbiol..

[23] Biddle J.F., Lipp J.S., Leverd M.A., Lloydd K.G., Sørensen K.B., Andersonc R., Fredricks H.F., Elvertc M., Kelly T.J., Schragh D.P. (2006). Heterotrophic Archaea dominate sedimentary subsurface ecosystems off Peru. Proc. Natl. Acad. Sci. USA.

[24] Inagaki F., Nunoura T., Nakagawa S., Teske A., Lever M., Lauer A., Suzuki M., Takai K., Delwiche M., Colwell F.S. (2006). Biogeographical distribution and diversity of microbes in methane hydrate-bearing deep marine sediments on the Pacific Ocean Margin. Proc. Natl. Acad. Sci. USA.

[25] Gan S., Schmidt F., Heuer V.B., Goldhammer T., Witt M., Hinrichs K.-U. (2020). Impacts of redox conditions on dissolved organic matter (DOM) quality in marine sediments off the River Rhône, Western Mediterranean Sea. Geochim. Cosmochim. Acta.

[26] Wakeham S.G., Canuel E.A. (2006). Degradation and Preservation of Organic Matter in Marine Sediments. Marine Organic Matter: Biomarkers, Isotopes and DNA.

[27] LaRowe D.E., Arndt S., Bradley J.A., Estes E.R., Hoarfrost A., Lang S.Q., Lloyd K.G., Mahmoudi N., Orsi W.D., Shah Walter S.R. (2020). The fate of organic carbon in marine sediments—New insights from recent data and analysis. Earth-Sci. Rev..

[28] Arnosti C. (2011). Microbial extracellular enzymes and the marine carbon cycle. Ann. Rev. Mar. Sci..

[29] Kamalanathan M., Doyle S.M., Xu C., Achberger A.M., Wade T.L., Schwehr K., Santschi P.H., Sylvan J.B., Quigg A. (2020). Exoenzymes as a Signature of Microbial Response to MarineEnvironmental Conditions. Appl. Environ. Sci..

[30] Bernardet J.-F., Bowman J.P. (2006). The Genus Flavobacterium. The Prokaryotes.

[31] Dyksma S., Lenk S., Sawicka J.E., Mussmann M. (2018). Uncultured Gammaproteobacteria and Desulfobacteraceae Account for Major Acetate Assimilation in a Coastal Marine Sediment. Front. Microbiol..

[32] Muller A.L., Pelikan C., de Rezende J.R., Wasmund K., Putz M., Glombitza C., Kjeldsen K.U., Jorgensen B.B., Loy A. (2018). Bacterial interactions during sequential degradation of cyanobacterial necromass in a sulfidic arctic marine sediment. Environ. Microbiol..

[33] Glöckner F.O., Kube M., Bauer M., Teeling H., Lombardot T., Ludwig W., Gade D., Beck A., Borzym K., Heitmann K. (2003). Complete genome sequence of the marine planctomycete Pirellula sp. strain 1. Proc. Natl. Acad. Sci. USA.

[34] Linz A.M., Crary B.C., Shade A., Owens S., Gilbert J.A., Knight R., McMahon K.D. (2017). Bacterial Community Composition and Dynamics Spanning Five Years in Freshwater Bog Lakes. mSphere.

[35] Dombrowski N., Teske A.P., Baker B.J. (2018). Expansive microbial metabolic versatility and biodiversity in dynamic Guaymas Basin hydrothermal sediments. Nat. Commun..

[36] Tully B.J., Graham E.D., Heidelberg J.F. (2018). The reconstruction of 2,631 draft metagenome-assembled genomes from the global oceans. Sci. Data.

[37] Parks D.H., Rinke C., Chuvochina M., Chaumeil P.A., Woodcroft B.J., Evans P.N., Hugenholtz P., Tyson G.W. (2017). Recovery of nearly 8000 metagenome-assembled genomes substantially expands the tree of life. Nat. Microbiol..

[38] Kantor R.S., Wrighton K.C., Handley K.M., Sharon I., Hug L.A., Castelle C.J., Thomas B.C., Banfield J.F. (2013). Small genomes and sparse metabolisms of sediment-associated bacteria from four candidate phyla. mBio.

[39] Luef B., Frischkorn K.R., Wrighton K.C., Holman H.Y., Birarda G., Thomas B.C., Singh A., Williams K.H., Siegerist C.E., Tringe S.G. (2015). Diverse uncultivated ultra-small bacterial cells in groundwater. Nat. Commun..

[40] Brown C.T., Hug L.A., Thomas B.C., Sharon I., Castelle C.J., Singh A., Wilkins M.J., Wrighton K.C., Williams K.H., Banfield J.F. (2015). Unusual biology across a group comprising more than 15% of domain Bacteria. Nature.

[41] Vigneron A., Cruaud P., Langlois V., Lovejoy C., Culley A.I., Vincent W.F. (2019). Ultra-small and abundant: Candidate phyla radiation bacteria are potential catalysts of carbon transformation in a thermokarst lake ecosystem. Limnol. Oceanogr. Lett..

[42] Guenet B., Danger M., Abbadie L., Lacroix G. (2010). Priming effect: Bridging the gap between terrestrial and aquatic ecology. Ecology.

[43] Osterholz H., Singer G., Wemheuer B., Daniel R., Simon M., Niggemann J., Dittmar T. (2016). Deciphering associations between dissolved organic molecules and bacterial communities in a pelagic marine system. ISME J..

[44] Osterholz H., Niggemann J., Giebel H.A., Simon M., Dittmar T. (2015). Inefficient microbial production of refractory dissolved organic matter in the ocean. Nat. Commun..

[45] Oni O.E., Schmidt F., Miyatake T., Kasten S., Witt M., Hinrichs K.U., Friedrich M.W. (2015). Microbial Communities and Organic Matter Composition in Surface and Subsurface Sediments of the Helgoland Mud Area, North Sea. Front. Microbiol..

[46] Ahrens J., Beck M., Marchant H.K., Ahmerkamp S., Schnetger B., Brumsack H.J. (2020). Seasonality of Organic Matter Degradation Regulates Nutrient and Metal Net Fluxes in a High Energy Sandy Beach. J. Geophys. Res. Biogeosci..

[47] Waska H., Simon H., Ahmerkamp S., Greskowiak J., Ahrens J., Seibert S.L., Schwalfenberg K., Zielinski O., Dittmar T. (2021). Molecular Traits of Dissolved Organic Matter in the Subterranean Estuary of a High-Energy Beach: Indications of Sources and Sinks. Front. Mar. Sci..

[48] Degenhardt J., Dlugosch L., Ahrens J., Beck M., Waska H., Engelen B. (2020). Seasonal Dynamics of Microbial Diversity at a Sandy High Energy Beach Reveal a Resilient Core Community. Front. Mar. Sci..

[49] Degenhardt J., Khodami S., Milke F., Waska H., Engelen B., Martinez Arbizu P. (2021). The Three Domains of Life Within the Discharge Area of a Shallow Subterranean Estuary at a High Energy Beach. Front. Environ. Sci..

[50] Wemheuer F., Taylor J.A., Daniel R., Johnston E., Meinicke P., Thomas T., Wemheuer B. (2020). Tax4Fun2: Prediction of habitat-specific functional profiles and functional redundancy based on 16S rRNA gene sequences. Environ. Microbiome.

[51] Grünenbaum N., Ahrens J., Beck M., Gilfedder B.S., Greskowiak J., Kossack M., Massmann G. (2020). A Multi-Method Approach for Quantification of In- and Exfiltration Rates from the Subterranean Estuary of a High Energy Beach. Front. Earth Sci..

[52] Grünenbaum N., Greskowiak J., Sültenfuß J., Massmann G. (2020). Groundwater flow and residence times below a meso-tidal high-energy beach: A model-based analyses of salinity patterns and 3H-3He groundwater ages. J. Hydrol..

[53] Waska H., Greskowiak J., Ahrens J., Beck M., Ahmerkamp S., Böning P., Brumsack H.J., Degenhardt J., Ehlert C., Engelen B. (2019). Spatial and Temporal Patterns of Pore Water Chemistry in the Inter-Tidal Zone of a High Energy Beach. Front. Mar. Sci..

[54] Itaya K., Ui M. (1966). A New Micromethod For The Colorimetric Determination of Inorganic Phosphate. Clin. Chim. Acta.

[55] Laskov C., Herzog C., Lewandowski J., Hupfe M. (2007). Miniaturized photometrical methods for the rapid analysis of phosphate, ammonium, ferrous iron, and sulfate in pore water of freshwater sediments. Limnol. Oceanogr. Methods.

[56] Merder J., Freund J.A., Feudel U., Hansen C.T., Hawkes J.A., Jacob B., Klaproth K., Niggemann J., Noriega-Ortega B.E., Osterholz H. (2020). ICBM-OCEAN: Processing Ultrahigh-Resolution Mass Spectrometry Data of Complex Molecular Mixtures. Anal. Chem..

[57] Lueders T., Manefield M., Friedrich M.W. (2004). Enhanced sensitivity of DNA- and rRNA-based stable isotope probing by fractionation and quantitative analysis of isopycnic centrifugation gradients. Environ. Microbiol..

[58] Gabor E.M., Vries E.J., Janssen D.B. (2003). Efficient recovery of environmental DNA for expression cloning by indirect extraction methods. FEMS Microbiol. Ecol..

[59] Legendre P., Legendre L. (2012). Numerical Ecology.

[60] Hahs-Vaughn D.L. (2016). Applied Multivariate Statistical Concepts.

[61] Dufrene M., Legendre P. (1997). Species Assemblages and Indicator Species: The Need for a Flexible Asymmetrical Approach. Ecol. Monogr..

[62] Benjamini Y., Hochberg Y. (1995). Controlling the False Discovery Rate: A Practical and Powerful Approach to Multiple Testing. J. R. Stat. Soc. Ser. B.

[63] Hothorn T., Hornik K., Zeileis A. (2006). Unbiased Recursive Partitioning: A Conditional Inference Framework. J. Comput. Graph. Stat..

[64] Quast C., Pruesse E., Yilmaz P., Gerken J., Schweer T., Yarza P., Peplies J., Glockner F.O. (2013). The SILVA ribosomal RNA gene database project: Improved data processing and web-based tools. Nucleic Acids Res..

[65] Kanehisa M., Goto S. (2000). KEGG: Kyoto Encyclopedia of Genes and Genomes. Nucleic Acids Res..

[66] Wickham H. (2011). ggplot2. Wiley Interdiscip. Rev. Comput. Stat..

[67] Auguie B., Antonov A., Auguie M.B. (2017). Package ‘gridextra’, Miscellaneous Functions for “Grid” Graphics. https://cran.rproject.org/web/packages/gridExtra/gridExtra.pdf.

[68] Collingro A., Toenshoff E.R., Taylor M.W., Fritsche T.R., Wagner M., Horn M. (2005). ‘Candidatus Protochlamydia amoebophila’, an endosymbiont of Acanthamoeba spp.. Int. J. Syst. Evol. Microbiol..

[69] Flerus R., Lechtenfeld O.J., Koch B.P., McCallister S.L., Schmitt-Kopplin P., Benner R., Kaiser K., Kattner G. (2012). A molecular perspective on the ageing of marine dissolved organic matter. Biogeosciences.

[70] D’Andrilli J., Cooper W.T., Foreman C.M., Marshall A.G. (2015). An ultrahigh-resolution mass spectrometry index to estimate natural organic matter lability. Rapid Commun. Mass Spectrom..

[71] Medeiros P.M., Seidel M., Niggemann J., Spencer R.G.M., Hernes P.J., Yager P.L., Miller W.L., Dittmar T., Hansell D.A. (2016). A novel molecular approach for tracing terrigenous dissolved organic matter into the deep ocean. Glob. Biogeochem. Cycles.

[72] D’Andrilli J., Junker J.R., Smith H.J., Scholl E.A., Foreman C.M. (2019). DOM composition alters ecosystem function during microbial processing of isolated sources. Biogeochemistry.

[73] Linkhorst A., Dittmar T., Waska H. (2017). Molecular Fractionation of Dissolved Organic Matter in a Shallow Subterranean Estuary: The Role of the Iron Curtain. Environ. Sci. Technol..

[74] Riedel T., Zark M., Vähätalo A.V., Niggemann J., Spencer R.G.M., Hernes P.J., Dittmar T. (2016). Molecular Signatures of Biogeochemical Transformations in Dissolved Organic Matter from Ten World Rivers. Front. Earth Sci..

[75] Mostovaya A., Hawkes J.A., Dittmar T., Tranvik L.J. (2017). Molecular Determinants of Dissolved Organic Matter Reactivity in Lake Water. Front. Earth Sci..

[76] Solden L., Lloyd K., Wrighton K. (2016). The bright side of microbial dark matter: Lessons learned from the uncultivated majority. Curr. Opin. Microbiol..

[77] Danczak R.E., Johnston M.D., Kenah C., Slattery M., Wrighton K.C., Wilkins M.J. (2017). Members of the Candidate Phyla Radiation are functionally differentiated by carbon- and nitrogen-cycling capabilities. Microbiome.

[78] Kojima H., Shinohara A., Fukui M. (2015). Sulfurifustis variabilis gen. nov., sp. nov., a sulfur oxidizer isolated from a lake, and proposal of Acidiferrobacteraceae fam. nov. and Acidiferrobacterales ord. nov. Int. J. Syst. Evol. Microbiol..

[79] Kolinko S., Richter M., Glockner F.O., Brachmann A., Schuler D. (2016). Single-cell genomics of uncultivated deep-branching magnetotactic bacteria reveals a conserved set of magnetosome genes. Environ. Microbiol..

[80] Dharamshi J.E., Tamarit D., Eme L., Stairs C.W., Martijn J., Homa F., Jorgensen S.L., Spang A., Ettema T.J.G. (2020). Marine Sediments Illuminate Chlamydiae Diversity and Evolution. Curr. Biol..

[81] Riedel T., Lettmann K., Schnetger B., Beck M., Brumsack H.-J. (2011). Rates of trace metal and nutrient diagenesis in an intertidal creek bank. Geochim. Cosmochim. Acta.

[82] Waska H., Brumsack H.-J., Massmann G., Koschinsky A., Schnetger B., Simon H., Dittmar T. (2019). Inorganic and organic iron and copper species of the subterranean estuary: Origins and fate. Geochim. Cosmochim. Acta.

[83] Krzmarzick M.J., Crary B.B., Harding J.J., Oyerinde O.O., Leri A.C., Myneni S.C., Novak P.J. (2012). Natural niche for organohalide-respiring Chloroflexi. Appl. Environ. Microbiol..

[84] Adrian L., Löffler F.E. (2016). Organohalide—Respiring Bacteria.

[85] Kaster A.K., Mayer-Blackwell K., Pasarelli B., Spormann A.M. (2014). Single cell genomic study of Dehalococcoidetes species from deep-sea sediments of the Peruvian Margin. ISME J..

[86] Biddle J.F., Fitz-Gibbon S., Schuster S.C., Brenchley J.E., House C.H. (2008). Metagenomic signatures of the Peru Margin subseafloor biosphere show a genetically distinct environment. Proc. Natl. Acad. Sci. USA.

[87] Wasmund K., Schreiber L., Lloyd K.G., Petersen D.G., Schramm A., Stepanauskas R., Jorgensen B.B., Adrian L. (2014). Genome sequencing of a single cell of the widely distributed marine subsurface Dehalococcoidia, phylum Chloroflexi. ISME J..

[88] Löffler F.E., Yan J., Ritalahti K.M., Adrian L., Edwards E.A., Konstantinidis K.T., Müller J.A., Fullerton H., Zinder S.H., Spormann A.M. (2013). Dehalococcoides mccartyi gen. nov., sp. nov., obligately organohalide-respiring anaerobic bacteria relevant to halogen cycling and bioremediation, belong to a novel bacterial class, Dehalococcoidia classis nov., order Dehalococcoidales ord. nov. and family Dehalococcoidaceae fam. nov., within the phylum Chloroflexi. Int. J. Syst. Evol. Microbiol..

[89] Wilms R., Sass H., Kopke B., Koster J., Cypionka H., Engelen B. (2006). Specific bacterial, archaeal, and eukaryotic communities in tidal-flat sediments along a vertical profile of several meters. Appl. Environ. Microbiol..

[90] Buchan A., LeCleir G.R., Gulvik C.A., Gonzalez J.M. (2014). Master recyclers: Features and functions of bacteria associated with phytoplankton blooms. Nat. Rev. Microbiol..

[91] Teeling H., Fuchs B.M., Becher D., Klockow C., Gardebrecht A., Bennke C.M., Kassabgy M., Huang S., Mann A.J., Waldmann J.T. (2012). Substrate-Controlled Succession of Marine Bacterioplankton Populations Induced by a Phytoplankton Bloom. Science.

[92] Fukunaga Y., Kurahashi M., Sakiyama Y., Ohuchi M., Yokota A., Harayama S. (2009). Phycisphaera mikurensis gen. nov., sp. nov., isolated from a marine alga, and proposal of Phycisphaeraceae fam. nov., Phycisphaerales ord. nov. and Phycisphaerae classis nov. in the phylum Planctomycetes. J. Gen. Appl. Microbiol..

[93] Ostgaard K. (1992). Enzymatic microassay for the determination and characterization of alginates. Carbohydr. Polym..

[94] Raeke J., Lechtenfeld O.J., Wagner M., Herzsprung P., Reemtsma T. (2016). Selectivity of solid phase extraction of freshwater dissolved organic matter and its effect on ultrahigh resolution mass spectra. Environ. Sci. Process. Impacts.

