# Supplementary material for: Cross-Shore and Depth Zonations in Bacterial Diversity Are Linked to Age and Source of Dissolved Organic Matter across the Intertidal Area of a Sandy Beach
Source: Microorganisms. 2021 Aug 12;9(8):1720. doi: 10.3390/microorganisms9081720 (PMC8399146; doi:10.3390/microorganisms9081720)
Supplement: Supplementary file 1 [file microorganisms-09-01720-s001.zip › Supplement_Degenhardt_et_al_2021b.pdf]

microorganisms

Supplements to:

Cross-shore and depth zonations in bacterial diversity are linked to age and source of dissolved organic matter across the intertidal area of a sandy beach

Julius Degenhardt[1], Julian Merder[2], Benedikt Heyerhoff[1], Heike Simon[1], Bert Engelen[1*], Hannelore Waska[1]

[1]Institute for Chemistry and Biology of the Marine Environment, University of Oldenburg, Oldenburg, Germany

[2]Department of Global Ecology, Carnegie Institution for Science, Stanford, CA, USA

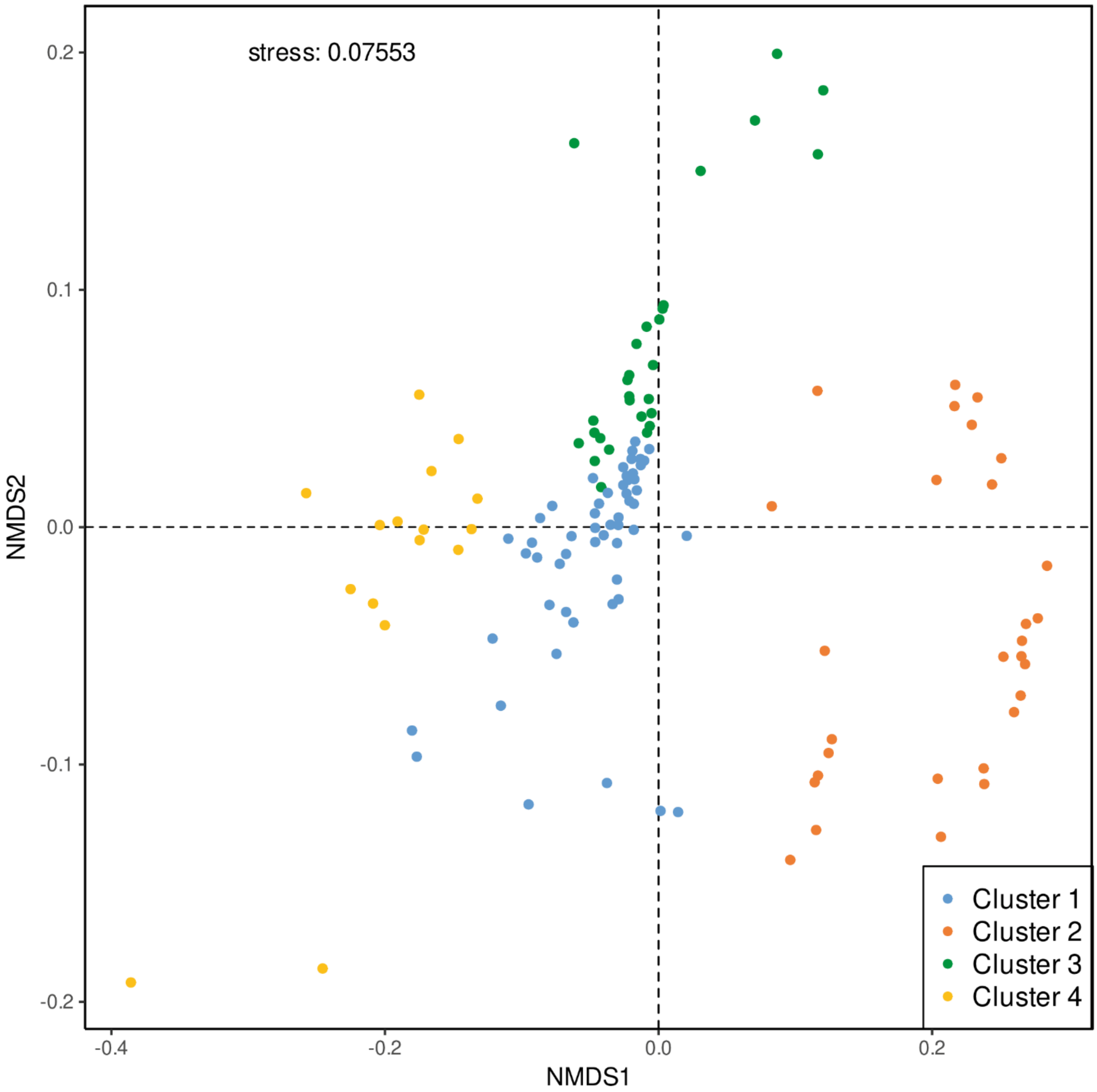

Figure S1: NMDS of all Hellinger standardized zOTUs of the respective clusters. Colors indicate cluster affiliation, distance between datapoints indicates taxonomic dissimilarity.

Table S1: Wilcoxson pairwise comparisons between environmental factors and DOM indices of each cluster. P- values are given for each significant difference.

	I v II	I v III	I v IV	II v III	II v IV	III v IV
Salinity						
O2_conc			p<0.05			p<0.05
NH4	p<0.001			p<0.001		
NO3	p<0.05			p<0.05		
Si	p<0.05	p<0.01		p<0.001	p<0.05	p<0.05
Fe	p<0.001			p<0.001		
Mn	p<0.001			p<0.001	p<0.05	
DOC	p<0.001			p<0.001	p<0.01	
FDOM	p<0.001	p<0.01		p<0.001	p<0.01	p<0.05
Homoseries	p<0.05					
H/C						
O/C						
N		p<0.05		p<0.01		
S	p<0.001			p<0.001		
mz	p<0.001			p<0.001		
AI.mod	p<0.05			p<0.05		
DBE						
Aromatic	p<0.001			p<0.001	p<0.05	
HU	p<0.001			p<0.001	p<0.01	
Unsaturated	p<0.05			p<0.05		
Saturated				p<0.05		
Ideg	p<0.001			p<0.001		
MLB_l						
Iterr	p<0.001			p<0.001	p<0.01	

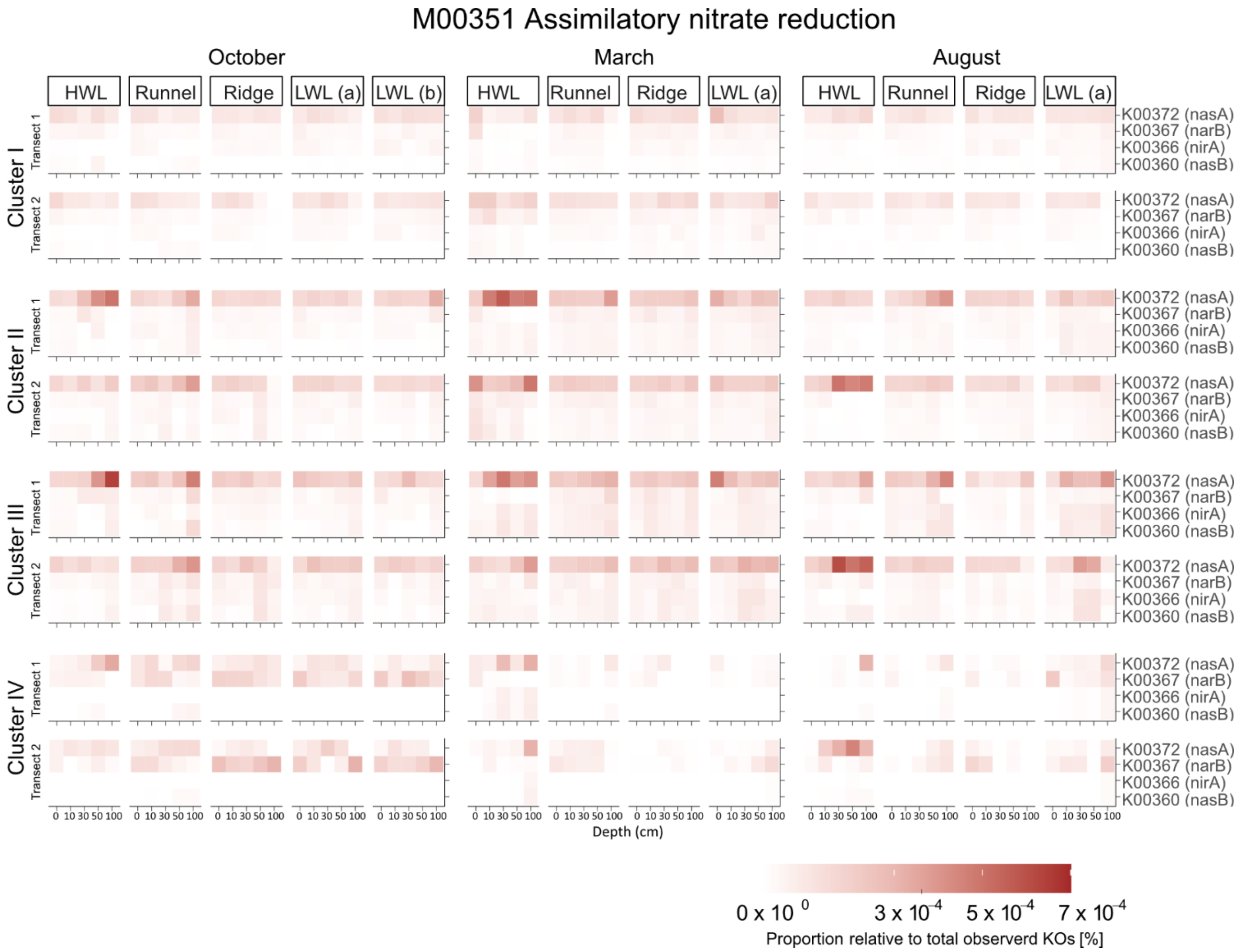

Figure S2: Predicted expression of genes involved in assimilatory nitrate reduction in relation to all KEGG orthologs (Kos) observed. Gene expression levels are presented individually for each of the four clusters at every station and depths and month across the two transects sampled.

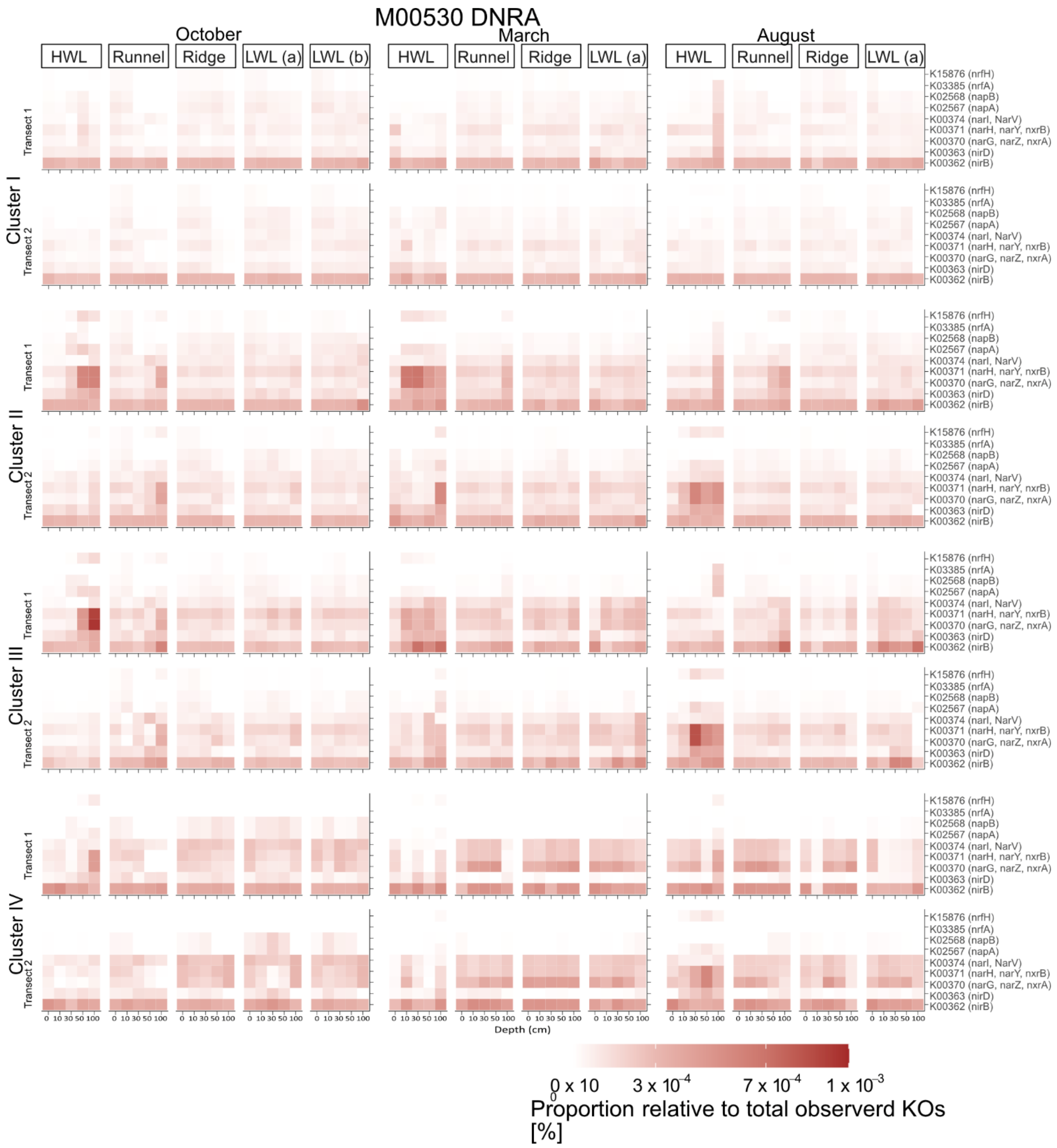

Figure S3: Predicted expression of genes involved in dissimilatory nitrate reduction to ammonium in relation to all KEGG orthologs (Kos) observed. Gene expression levels are presented individually for each of the four clusters at every station and depths and month across the two transects sampled.

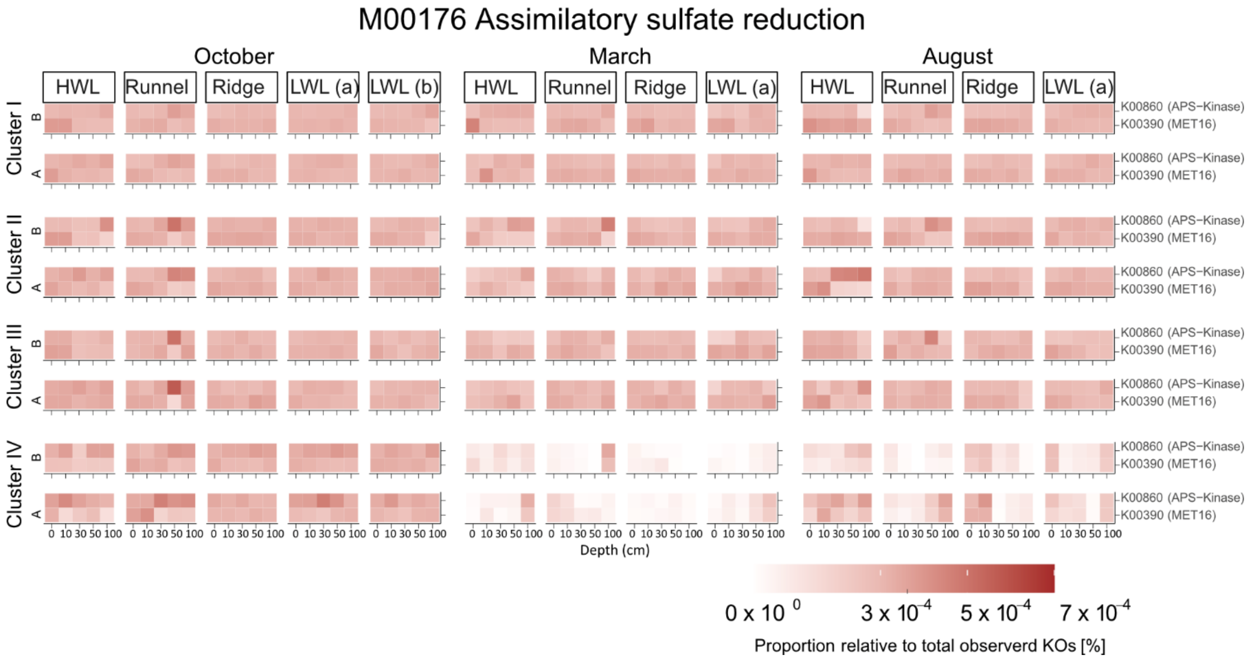

Figure S4: Predicted expression of genes involved in assimilatory sulfate reduction in relation to all KEGG orthologs (Kos) observed. Gene expression levels are presented individually for each of the four clusters at every station and depths and month across the two transects sampled.

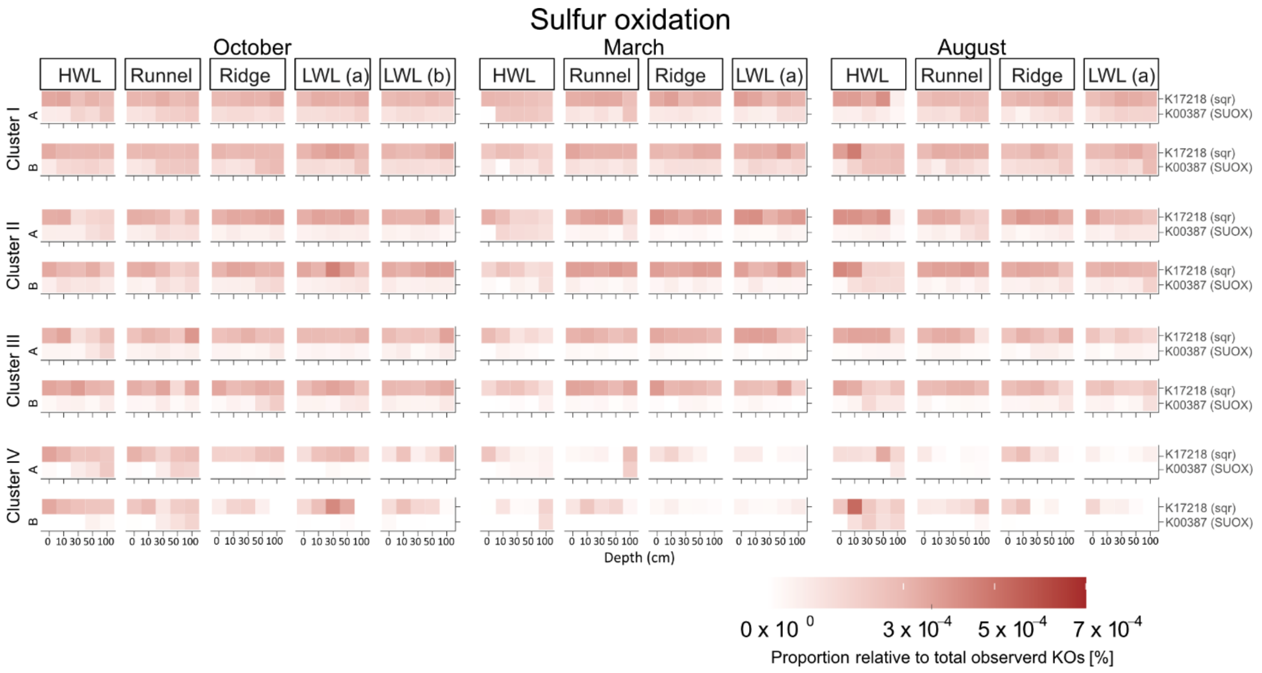

Figure S5: Predicted expression of genes involved in sulfur oxidation in relation to all KEGG orthologs (Kos) observed. Gene expression levels are presented individually for each of the four clusters at every station and depths and month across the two transects sampled.

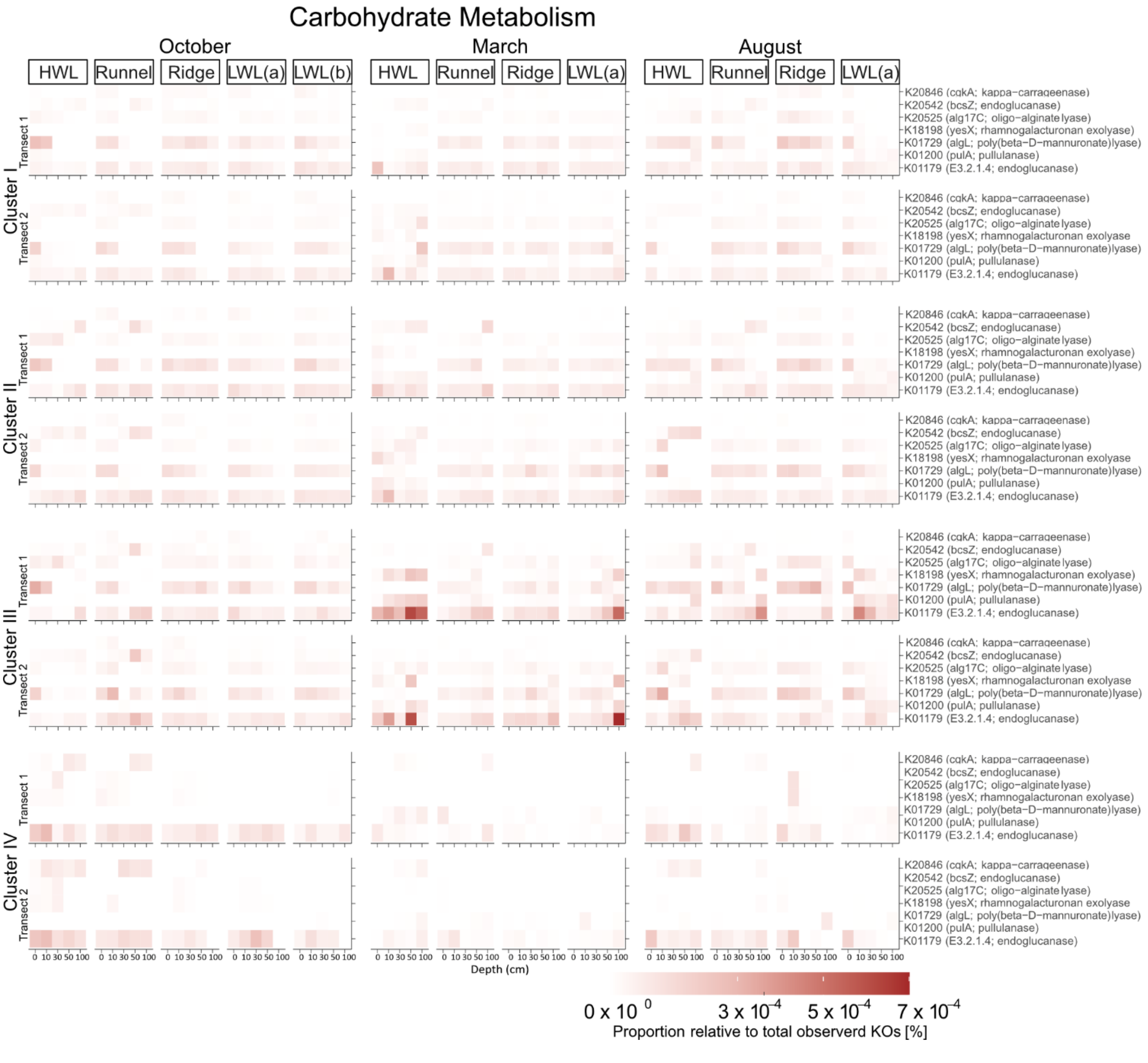

Figure S6: Predicted expression of genes involved the degradation of algal derived polymers in relation to all KEGG orthologs (Kos) observed. Gene expression levels are presented individually for each of the four clusters at every station and depths and month across the two transects sampled.

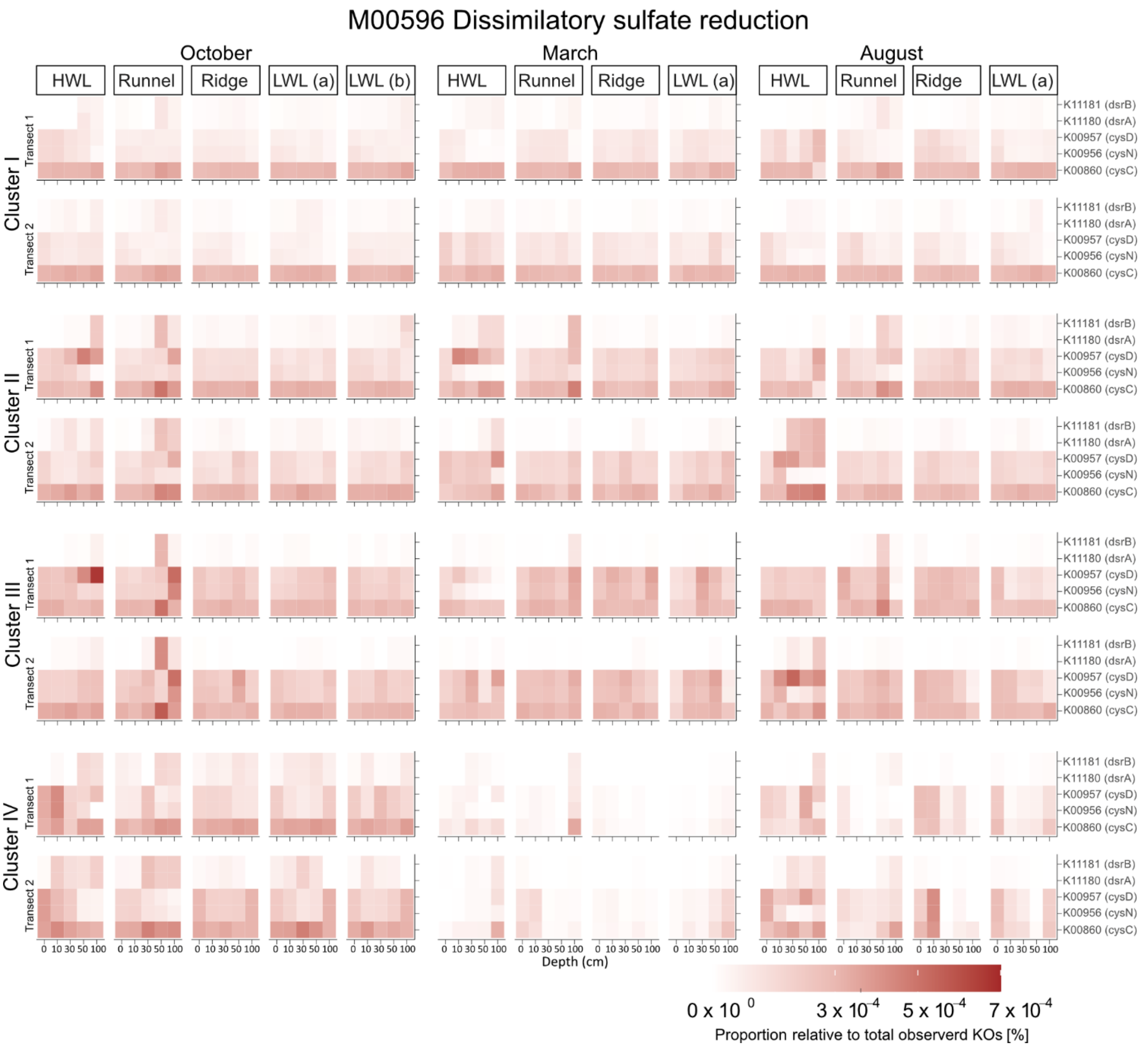

Figure S7: Predicted expression of genes involved in dissimilatory sulfate reduction in relation to all KEGG orthologs (Kos) observed. Gene expression levels are presented individually for each of the four clusters at every station and depths and month across the two transects sampled.

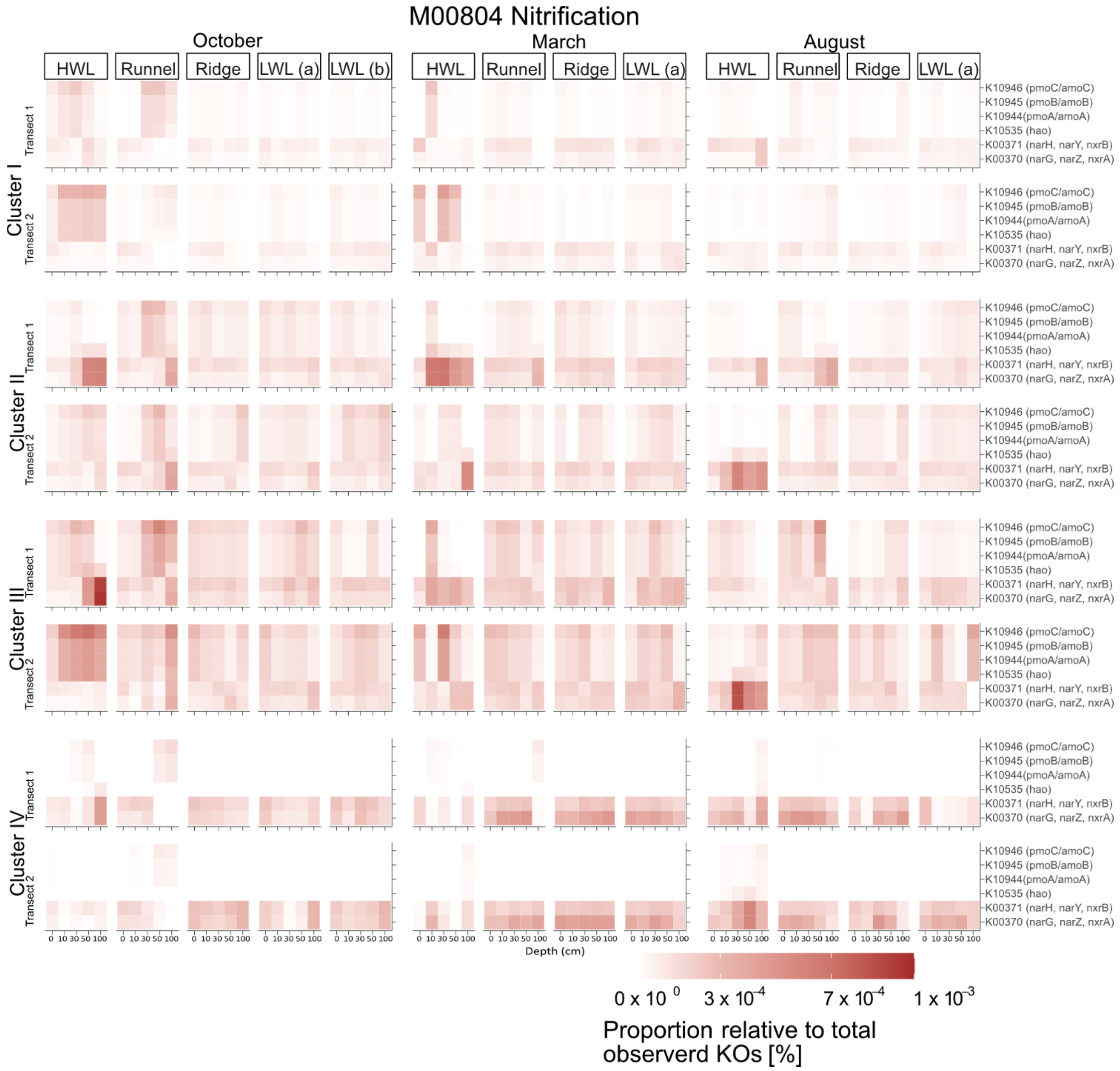

Figure S8: Predicted expression of genes involved in nitrification in relation to all KEGG orthologs (Kos) observed. Gene expression levels are presented individually for each of the four clusters at every station and depths and month across the two transects sampled.
